# Assessment of Paratuberculosis Vaccination Effect on In Vitro Formation of Neutrophil Extracellular Traps in a Sheep Model

**DOI:** 10.3390/vaccines10091403

**Published:** 2022-08-26

**Authors:** Noive Arteche-Villasol, Daniel Gutiérrez-Expósito, Miguel Criado, Julio Benavides, Valentín Pérez

**Affiliations:** 1 Departamento de Sanidad Animal, Facultad de Veterinaria, Campus de Vegazana, Universidad de León, 24071 León, Spain; 2 Departamento de Sanidad Animal, Instituto de Ganadería de Montaña (CSIC-ULE), Finca Marzanas, Grulleros, 24346 León, Spain

**Keywords:** vaccination, paratuberculosis, sheep, NETs

## Abstract

Vaccination of domestic ruminants against paratuberculosis has been related to homologous and heterologous protective effects that have been attributed to the establishment of a trained immune response. Recent evidence suggests that neutrophils could play a role in its development. Therefore, we propose an in vitro model for the study of the effect of paratuberculosis vaccination on the release of neutrophil extracellular traps (NETs) in sheep. Ovine neutrophils were obtained from non-vaccinated (*n* = 5) and vaccinated sheep (*n* = 5) at different times post-vaccination and infected in vitro with *Mycobacterium avium* subsp. *paratuberculosis* (*Map*), *Staphylococcus aureus* (*SA*), and *Escherichia coli* (*EC*). NETs release was quantified by fluorimetry and visualized by immunofluorescence microscopy. Typical NETs components (DNA, neutrophil elastase, and myeloperoxidase) were visualized extracellularly in all infected neutrophils; however, no significant percentage of extracellular DNA was detected in *Map-*infected neutrophils compared with *SA*- and *EC*-infected. In addition, no significant effect was detected in relation to paratuberculosis vaccination. Further assays to study NETs release in ovine neutrophils are needed. Preliminary results suggest no implication of NETs formation in the early immune response after vaccination, although other neutrophil functions should be evaluated.

## 1. Introduction

Paratuberculosis is a chronic debilitating disease of ruminants caused by the intracellular pathogen *Mycobacterium avium* subsp. *paratuberculosis* (*Map*) that causes substantial economic losses worldwide [1]. Vaccination with approved vaccines has resulted in a reliable and cost-efficient tool to reduce the onset of the clinical disease in field conditions [2,3]. Nevertheless, the response of vaccinated animals is not homogeneous, and some animals could develop severe intestinal lesions without an explanation being found, since the mechanisms by which vaccination ensure protection are unclear [4,5].

Macrophages are the niche where *Map* survive and multiply [6]. Therefore, numerous studies have focused on the interaction between *Map* and macrophages [7,8,9]. In contrast, little is known about the possible involvement of neutrophils during paratuberculosis pathogenesis, due to their short lifespan and the unavailability of the mycobacteria sheltered inside macrophages. Nonetheless, some studies have reported that *Map* infection could induce an early migration of neutrophils to the infection site and a malfunction during neutrophil recruitment [10,11,12]. Indeed, it has been shown that bovine neutrophils are able to release NETs, a purely extracellular antimicrobial mechanism, in response to the in vitro infection with *Map* [13].

In addition, unlike the growing number of studies that assess the effect of vaccination on the interaction between macrophages and *Map* [14,15], the role of neutrophils in the *Map* vaccination-induced protective response has been scarcely investigated. Recent evidence has shown that infection of neutrophils from paratuberculosis-vaccinated rabbits results in enhanced responsiveness (i.e., phagocytosis, NETs release, etc.) of these cells against *Map* and other non-mycobacterial pathogens [16]. This behavior has been related to the establishment of a “trained immunity”, characterized by the arbitration of innate immune cells whose function has been altered due to a long-term reprogramming [17]. This immune response has typically been associated with macrophages, due to their long lifespan, although some studies concurred that neutrophils might undergo similar epigenetic changes after vaccination [17,18].

Due to the limited comprehension about the mechanisms implicated in the protective immune response elicited by paratuberculosis vaccination, in the current study we investigated for the first time whether paratuberculosis vaccination has any influence on NETs generation in ovine neutrophils infected in vitro with different pathogens.

## 2. Materials and Methods

### 2.1. Animals

A total of ten healthy one-year-old female sheep of the Rasa Aragonesa breed were selected from the experimental flock of the Instituto de Ganadería de Montaña (CSIC-ULE) in Grulleros, León. All sheep were *Map*-free, confirmed by an indirect enzyme-linked immunosorbent assay (ID Screen^®^ Paratuberculosis indirect, IDVet, Grabels, France) and interferon gamma (IFN-γ) release test (Bovigam^®^ *Mycobacterium bovis* IFN-γ test for cattle, Thermo Fisher Scientific AG, Basel, Switzerland) [19]. Animals were randomly divided into vaccinated (*n* = 5) and non-vaccinated (*n* = 5) groups, where the former was subcutaneously vaccinated with 1 mL of Silirum^®^ (CZ Vaccines, Porriño, Spain), whereas the non-vaccinated group was inoculated with 1 mL of phosphate buffered saline (PBS) by the same route. All animals were sampled the day before vaccination as well as 15 and 30 days post-vaccination (dpv). Heparinized blood was collected in each sampling to isolate neutrophils and carry out two in vitro assays.

### 2.2. Ovine Neutrophils’ Isolation and Culture

Ovine neutrophils were isolated as previously described [20]. Neutrophils were resuspended in RPMI 1640 medium without phenol red (Gibco^®^, Paisley, UK) and adjusted at a final concentration of 10^6^ cells mL^−1^. Cell viability (96%) and purity (90%) were determined by trypan blue exclusion and Diff-Quick staining, respectively. A total of 2 × 10^5^ neutrophils per well were seeded in 96-well flat-bottom plates (Thermo Fisher Scientific, Rochester, NY, USA) to perform neutrophil extracellular DNA quantification. In addition, an aliquot of 2 × 10^5^ neutrophils from each animal was prepared separately for genomic DNA extraction to calculate the percentage of NETs release. To carry out NETs visualization, 10^6^ neutrophils per well were seeded on sterile round glass coverslips of 13 mm diameter (VWR, Darmstadt, Germany), pretreated with 0.01% poly-L-lysine (EMD Millipore Corp, Darmstadt, Germany) in 24-well culture plates (Thermo Fischer Scientific, Rochester, NY, USA) [21]. For both in vitro assays, neutrophils were allowed to set for 1 h at 37 °C and 5% CO_2_ in a humidified incubator before infection.

### 2.3. Bacteria Culture and Infection

Live *Map* K10 reference strains, *SA* and *EC,* kindly provided by NEIKER (Basque Institute for Agricultural Research and Development, Derio, Spain), were prepared as previously described elsewhere [16]. After estimation by optical density (600 nm) and colony count on 7H9 broth, supplemented with oleic acid-albumin-dextrose-catalase enrichment (OADC) and mycobactin J (7H9-OADC-MJ) (*Map*), as well as brain-heart infusion (BHI) (*SA* and *EC*) agar using 10-fold serial dilutions, bacterial suspensions were adjusted and aliquoted at a concentration of 10^8^ colony-forming units (CFU) per mL in glycerol:water (1:1) and frozen at −80 °C until required.

Prior to neutrophil infection, aliquots were thawed in fresh 7H9-OADC-MJ (*Map*) and BHI (*SA* and *EC*) mediums and incubated for 3 h at 37 ± 1 °C [15]. Then, bacterial suspensions were centrifuged at 3000× *g* for 10 min and washed with PBS. Finally, bacterial pellets were resuspended in 1 mL of RPMI 1640 medium without phenol red and passed up and down through a 30-gauge needle to disperse the clumps before infection [15].

For both in vitro assays, neutrophils were infected in duplicate with *Map*, *SA,* and *EC* at a multiplicity of infection (MOI) of 10:1 (10 bacteria/1 neutrophil). Neutrophils stimulated in duplicate with 1 mg mL^−1^ of Zymosan (Sigma-Aldrich, St. Louis, MO, USA) were used as positive controls [22], whereas neutrophils with RPMI 1640 medium without phenol red served as negative controls. Afterwards, neutrophils were incubated for 4 h at 37 °C and 5% CO_2_ in a humidified incubator.

### 2.4. NETs Visualization

Neutrophils were washed twice with warm PBS and fixed with 1% CellFix (BD, Erembodegemdorp, Belgium) for 20 min at 37 °C. Thereupon, neutrophils were permeabilized with 0.1% Triton X-100 (Panreac, Barcelona, Spain) for 10 min and blocked with 5% bovine serum albumin (BSA) (Roche, Mannheim, Germany) for 1 h at 37 °C. For fluorescence staining, neutrophils were incubated with anti-neutrophil elastase (NE) primary antibody (ab68672, Abcam, Cambridge, UK) (1:200 in Animal-Free Blocker and Diluent, R.T.U, Vector Laboratories, Newark, CA, USA) for 1 h at 4 °C. Neutrophils were washed twice with PBS and secondary antibody goat anti-rabbit IgG Alexa Fluor^®^ 594 (ab150088, Abcam, Cambridge, UK) (1:1000) was added. Cells were kept in the dark for 1 h at 4 °C and washed twice with PBS. Then, neutrophils were incubated with anti-myeloperoxidase (MPO) primary antibody conjugated with Alexa Fluor^®^ 488 (bs-4943R-A488, Bioss Antibodies, Woburn, MA, USA) (1:400) for 1 h at 37 °C. Afterwards, cells were gently washed twice with PBS and mounted on glass slides using a mounting solution with DAPI medium (Abcam, Cambridge, UK). NETs visualization was carried out at 600× magnification on a direct fluorescence microscope (Eclipse Ni-E, Nikon, Melville, NY, USA) using appropriate epifluorescence filters. Images were captured using a CMOS scientific camera (Photometrics^®^ Prime BSI™, Scottsdale, AZ, USA).

### 2.5. Quantification of Extracellular DNA Using PicoGreen^®^

Neutrophil extracellular DNA was quantified as described elsewhere with few modifications [13]. After the incubation period, 0.1 U µL^−1^ of micrococcal nuclease (New England BioLabs, Ipswich, MA, USA) was added to the neutrophils seeded in 96-well plates and incubated for 15 min at 37 °C to disrupt neutrophil extracellular DNA. Afterwards, 5 mM of EDTA was incorporated to stop the nuclease activity and plates were centrifuged at 300× *g* for 15 min at 4 °C. Extracellular DNA was quantified in the supernatant using the Quant-iT™ PicoGreen^®^ kit (Invitrogen, Carlsbad, CA, USA) following the manufacturer’s instructions. Fluorescence was determined by spectrofluorometric analysis (Ex: 488 nm; Em: 520 nm) using an automated microplate reader (Biotek^®^ Synergy HT, Agilent Biotek, Santa Clara, CA, USA). Total DNA from a non-stimulated aliquot of 2 × 10^5^ neutrophils from each animal was extracted using the Maxwell^®^ 16 Cell DNA Purification Kit with the Maxwell 16 Instrument (Promega, Madison, WI, USA) following the manufacturer’s protocol, and fluorescence intensity was measured as mentioned above. Then, the percentage of extracellular DNA was calculated by dividing the fluorescence intensity of each sample by the fluorescence intensity of the total homologous genomic DNA [13].

### 2.6. Statistical Analysis

Normal distribution of DNA quantification was assessed using the Shapiro–Wilk test for small sample sizes. The generalized linear model (GLM) with binomial distribution was used to analyze the effect of vaccination, in vitro infection, and time post-vaccination on the log-transformed percentage of neutrophil extracellular DNA release. Then, comparisons were performed using the Student’s *t* test with the Tukey–Kramer adjustment for multiple comparisons. For all analyses, a *p*-value of <0.05 was statistically significant. All statistical analyses were performed with the R Software 4.0.3 (R Development Core Team, R Foundation for Statistical Computing, Vienna, Austria).

## 3. Results

### 3.1. NETs Visualization

**Figure 1:**
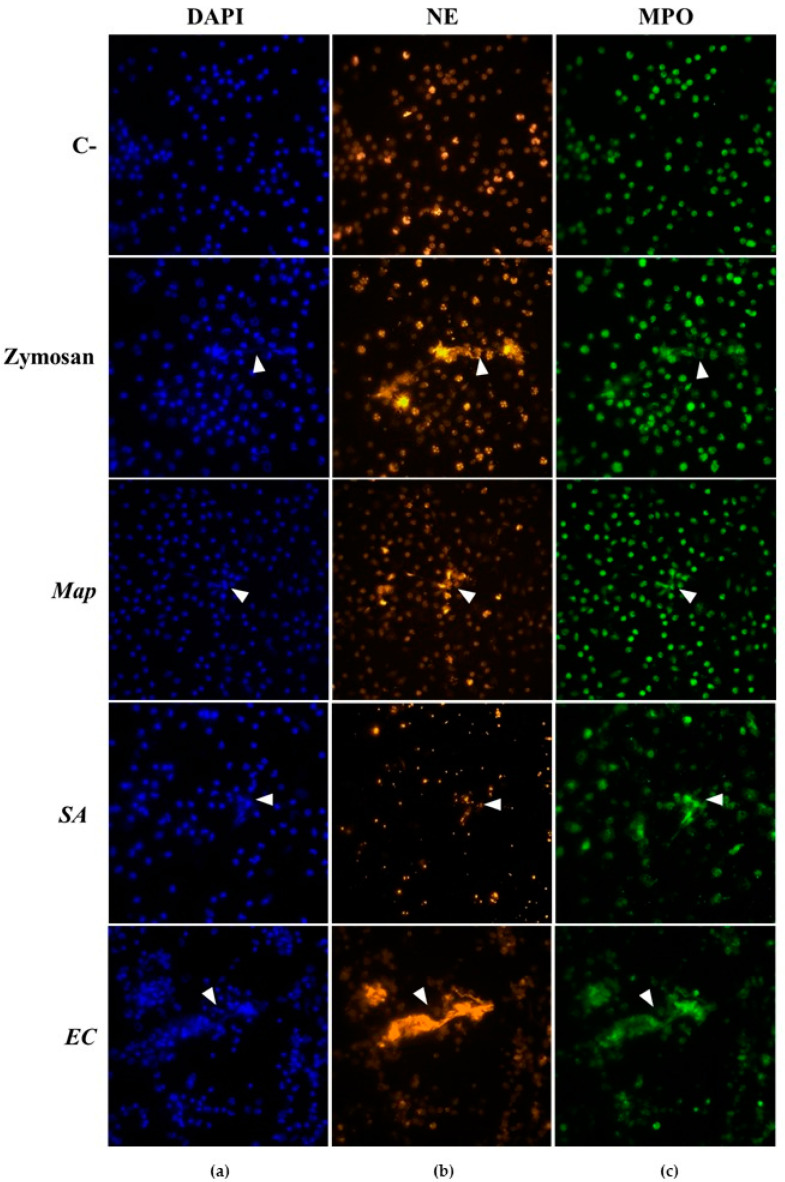
Visualization of neutrophil extracellular traps (NETs) in non-stimulated (C-), Zymosan-stimulated (Zymosan), and *Mycobacterium avium* subsp. *paratuberculosis-* (*Map*), *Staphylococcus aureus-* (*SA*), and *Escherichia coli* (*EC*)-infected neutrophils. NETs were composed by (**a**) DNA (DAPI; blue), (**b**) neutrophil elastase (NE; orange), and (**c**) myeloperoxidase (MPO; green). White triangular arrowheads point out to NET-like structures. Photomicrographs (60×) are representative of both groups (vaccinated and non-vaccinated) and post-vaccination days since no differences were observed between these variables.

NETs generation was checked using immunofluorescence staining through the visualization of DNA, NE, and MPO (Figure 1). Non-stimulated neutrophils showed a compact, multilobed, segmented nucleus visible after DAPI staining, along with the presence of MPO and NE inside the intact cytoplasm (Figure 1). Besides, the nucleus of neutrophils stimulated with Zymosan had a similar appearance to those non-stimulated, although some neutrophils showed NETs, recognized as extracellular structures similar to strands of different sizes stained with DAPI, NE, and MPO (Figure 1). Regarding neutrophils infected with bacteria, few NET-like structures were observed in *Map*-infected neutrophils whose nucleus remained intact (Figure 1). In contrast, different grades of nuclear swelling and rupture were noticed in neutrophils after infection with *SA* and *EC*. In this sense, neutrophil cultures infected with *SA* showed a great quantity of disrupted cells releasing DNA and MPO into the extracellular space (Figure 1). Some neutrophils seemed to induce NETs, identified as fibers composed of DNA and MPO; however, in these cultures, NE staining could not be clearly observed due to the non-specific interferences with *SA* (Figure 1). Besides, a great number of aggregated neutrophils were visualized in *EC*-infected cultures, although NET-like structures were clearer than in *SA*-infected cultures, stained with DAPI, MPO, and NE (Figure 1). These observations were similar both in vaccinated and non-vaccinated sheep and at all times tested.

### 3.2. Extracellular DNA Quantification

**Figure 2:**
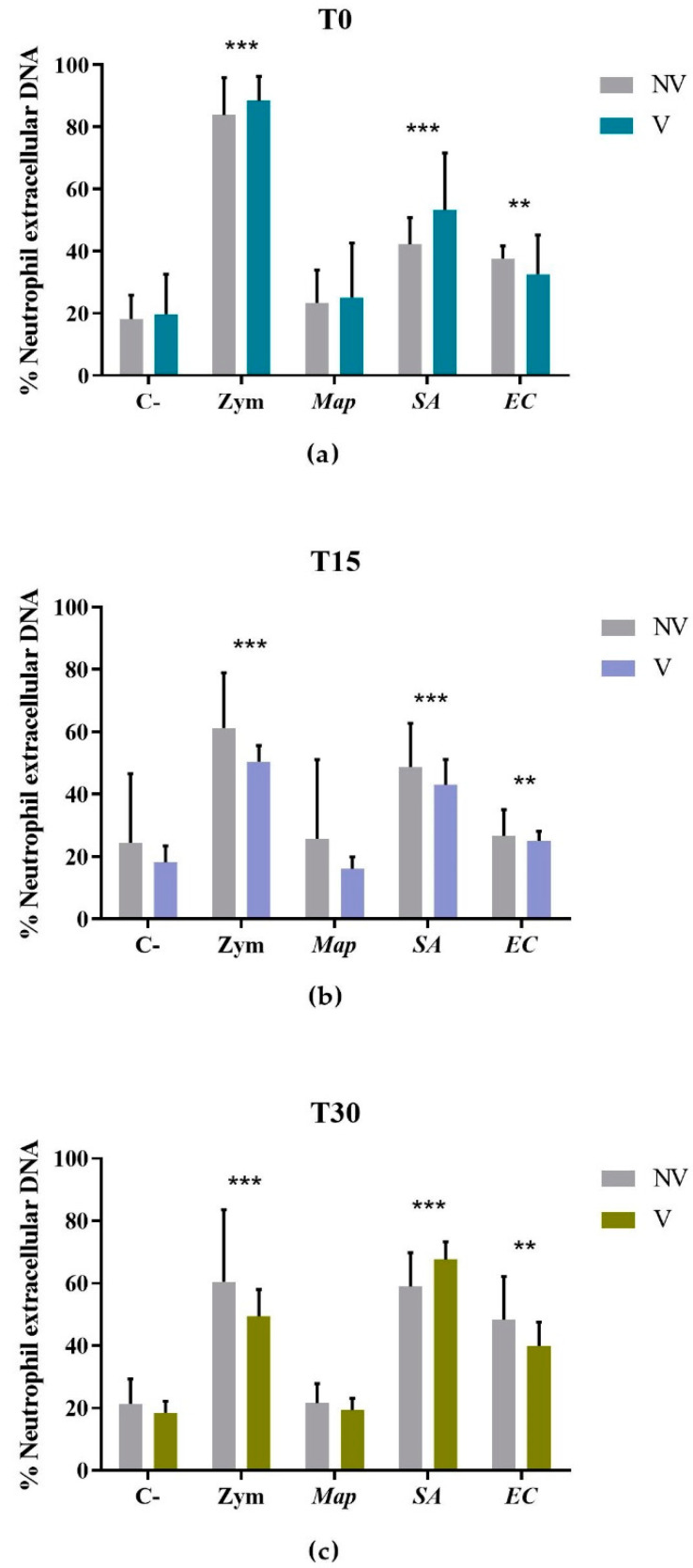
Percentage of extracellular DNA estimated by fluorometric quantification in non-vaccinated (NV) and vaccinated (V) groups. Bars and vertical lines represent mean percentage values and standard deviations, respectively, of non-stimulated (C-), Zymosan-stimulated (Zym), and *Mycobacterium avium* subsp. *paratuberculosis-* (*Map*), *Staphylococcus aureus-* (*SA*), and *Escherichia coli* (*EC*)-infected neutrophils (**a**) before vaccination (T0) and (**b**) 15 (T15) and (**c**) 30 days (T30) post-vaccination. Significant differences estimated by the generalized linear model were expressed as * *p* < 0.05, ** *p* < 0.01, *** *p* < 0.001, and **** *p* < 0.0001.

The GLM showed that the infection with *SA* (*p* < 0.001), *EC* (*p* < 0.01), and Zymosan (*p* < 0.001) had a clear effect in the release of extracellular DNA before vaccination as well as at 15 and 30 dpv, although the time of sampling or vaccination did not show any impact (*p* > 0.05) (Figure 2).

Multiple comparison analysis showed that infection with *SA* (52.44% ± 13.83%) produced a greater extracellular DNA release than non-stimulated (17.34% ± 5.60%) and *Map*-infected neutrophils (22.79% ± 12.39%) before vaccination (*p* < 0.01) and at 15 dpv (*p* < 0.0001) and 30 dpv (*p* < 0.0001). Furthermore, the percentage of extracellular DNA was significantly higher in *SA*-infected neutrophils than *EC*-infected (34.51% ± 11.20%) at 15 dpv (*p* < 0.001) and 30 dpv (*p* < 0.0001). Besides, a greater percentage of extracellular DNA was detected in neutrophils infected with *EC* in comparison to those neutrophils non-stimulated or infected with *Map* only at 30 dpv (*p* < 0.0001). In contrast, despite the slight increase observed in *Map*-infected neutrophils, no significant differences were observed compared to those that were non-stimulated at any time point (*p* > 0.05).

## 4. Discussion

Classically, neutrophils have been ruled out from the pathogenesis studies of numerous intracellular pathogens due to the inaccessibility of the latter. However, neutrophils have been demonstrated to play a fundamental role during pathogenic mycobacterial diseases, preventing their multiplication and dissemination during early stages of infection [23]. Regarding paratuberculosis disease, there is limited research about the possible involvement of neutrophils in the protection against *Map* infection [10,12,13]. Despite this fact, it has been proven that neutrophils could not only contribute to *Map* clearance, but also participate in the establishment of the protective immune response after vaccination against mycobacteria [13,16]. Thus, in this study, we investigated the early in vitro effect of paratuberculosis vaccination on ovine neutrophils by studying the ability of these cells to release NETs.

The function of NETs during paratuberculosis pathogenesis has not been fully elucidated. In this study, infection of ovine neutrophils with *Map* promoted the release of NETs, similar to that experienced in bovine neutrophils [13]. Nevertheless, when comparing this production with that observed in neutrophils infected with *SA* or *EC*, induction of NETs was significantly lower in *Map*-infected neutrophils. *SA* [24] and *EC* [25] seem to be strong inducers of NETs. For instance, *SA* infection has been shown to promote a rapid extracellular DNA release in human neutrophils within the first ten minutes that increased dramatically after four hours, at which time the production of NETs reaches its maximum and lysis of neutrophils begins to be appreciated [26]. This fact supports the findings observed here where a great number of neutrophils were disrupted, which could also explain the highest percentage of extracellular DNA.

The release of NETs is characterized by the immobilization and elimination of pathogens [27]. However, NETs have been reported to be unable to kill *M. tuberculosis* and their role may be more related to preventing its spread and stimulating granuloma formation [28]. A similar action could happen in paratuberculosis. The results of this study show that the generation of NETs after *Map* infection might not be a keynote mechanism, so its role would be limited to the tethering and the elimination of *Map*, enhancing its susceptibility to other antimicrobial mechanisms not evaluated in this study.

Regarding the effect of paratuberculosis vaccination, no differences were observed in the generation of NETs, at least not during the first month after vaccination. These results dissent from those obtained in rabbits, in which an increase of NETs release against live *Map* was detected in a pure neutrophil culture at three months after subcutaneous and oral vaccination with a similar inactivated paratuberculosis vaccine [16]. However, leaving aside differences in the immune response between rabbits and ruminants, our study analyzed the effect on NET formation only during the first month, and it is possible that changes may appear later. In addition, it is possible that the low number of animals per group (*n* = 5) hindered the detection of statistical differences between both groups. Besides, in contrast to our findings, an increase of the antimicrobial response has also been observed against live *M. bovis*, *Corynebacterium pseudotuberculosis*, *SA,* and *EC* in paratuberculosis-vaccinated rabbits, suggesting a possible training effect on neutrophils [16]. This effect was also observed in humans after Bacillus Calmette-Guérin (BCG) vaccination, showing a heterologous enhanced protection against non-related pathogens such as *Candida albicans*, though, in that study, NETs release was not affected by vaccination [18]. Thus, it is tempting to hypothesize that the protection associated with vaccination is not specifically linked to NETs but to other antimicrobial mechanisms oriented towards the improvement of phagocytosis by trained immunity or the activation of the adaptive immune response [29]. Considering these preliminary results, the quantification and visualization of NETs and the times post-vaccination evaluated here may not be sufficient to evaluate the effect of vaccination on neutrophils’ immune response. Therefore, other in vitro assays, to identify the formation of NETs and to determine other antimicrobial mechanisms, and longer times post-vaccination should be explored.

## Data Availability

Not applicable.
